# Supplementary material for: Learning curve and surgical outcome of robotic assisted colorectal surgery with ERAS program
Source: Sci Rep. 2022 Nov 29;12:20566. doi: 10.1038/s41598-022-24665-w (PMC9709162; doi:10.1038/s41598-022-24665-w)
Supplement: Supplementary file 1 — Supplementary Information 1. [file 41598_2022_24665_MOESM1_ESM.zip › LSC Raw data-Ñ[▒K/097/P(4)_ñw╝╨░O▒Kñσ.pdf]

[redacted] 及病理診斷中心 檢驗(查)報告

0938D 94002045

申請時間：2020/10/15 13:23 簽收時間：2020/10/15 14:26 檢體：Pathology
診斷醫師：文美卿 胡仔璇 報告時間：2020/10/22 16:25 病理號：S0932493

Pathologic diagnosis:
1. Rectum and anus, APR --- Status post neoadjuvant therapy with residual small focus of superficially invasive squamous cell carcinoma and high grade squamous intraepithelial lesion of anus. The bilateral cut ends, rectum and perirectal lymph nodes (0/7) are free of tumor.
2. Soft tissue, pelvis, dissection --- Unremarkable fibroadipose and muscular tissue.

Ancillary study for diagnosis:
Recut and IHC stains for AE1/AE3 (- on target lesion) and p16 (- on target lesion) done on section J; recut and P16 (+ on target lesion)IHC stain done on section E.

Prognostic and predictive factor:
1. Tumor size: 0.8 cm.
2. Depth of invasion: submucosa.
3. Histologic grade: well differentiated.
4. Angiolymphatic invasion: Not identified.
5. Perineural invasion: Not identified.
6. Tumor deposits (discontinuous extramural extension): Not identified.
7. Macroscopic tumor perforation: Not identified.
8. Surgical Margins: a) Proximal Margin: Uninvolved by invasive carcinoma. b) Distal Margin: Uninvolved by invasive carcinoma / HSIL, distance of tumor from margin: 2 mm. c) Radial or Mesenteric Margin: Uninvolved by invasive carcinoma.
9. Lymph node status: Uninvolved (0/7).
10. Extranodal involvement: Not identified.
11. Tumor regression grading S/P CCRT: Present; single cells or rare small groups of cancer cells (near complete response, score 1)
12. Type of polyp in which invasive carcinoma arose: Not identified.
13. Additional pathologic findings: high grade squamous intraepithelial lesion.
14. Pathological TNM Stage: ypT1N0(According to the eighth edition, American Joint Committee on Cancer Staging Guidelines for Tumors).
15. TNM descriptors: y (Post-treatment).

Gross description:
The specimen consists of 1) a segment of rectum and anus. The rectum is 17 cm in length and 2.5 cm in bilateral cut end circumference, and perirectal soft tissue. The anal tissue is 2.5x1.5x1 cm. A gray tan firm annular elevated and centrally ulcerative lesion, 3x3 cm, is noted at the anorectal junction, 1 cm to the distal cut end. The both cut ends are free of tumor grossly. 2) "pelcis", two pieces of tan yellow soft tissue, up to 3.5x2x0.5 cm. All for section: A) specimen 2; Representative part for section: B) proximal cut end C) lesion and distal cut end D+E) lesion at anorectal junction F-J) lesion with CFM K-U) lymph nodes.
#T-59600_2 #M-38000_2 0 1105 SP0000
#T-02508_2 #M-74003_2 0 1105 000000
#T-C4560_2 #M-09183_2 0 1105 000000

已電子簽章

敬告：本單僅供醫師參考使用，不具任何申請效力！！
請病患妥為保管，若不慎遺失需自負個人資料外洩責任！
